# Supplementary material for: Episodic Future Thinking about Smoking-Related Illness: A Preliminary Investigation of Effects on Delay Discounting, Cigarette Craving, and Cigarette Demand
Source: Int J Environ Res Public Health. 2022 Jun 10;19(12):7136. doi: 10.3390/ijerph19127136 (PMC9223435; doi:10.3390/ijerph19127136)
Supplement: Supplementary file 1 [file ijerph-19-07136-s001.zip › ijerph-1729882-supplementary.pdf]

Supplementary Table S1. *Two-Way Analysis of Covariance of Discounting AUC, Demand Measures, Craving, and Affect by Episodic Future Thinking and Smoking-Related Illness Groups.*

Variable	Source	*df*	*F*	*p*
Discounting rate (AUC)	Corrected model	4	3.883	0.005
	Intercept	1	362.729	0.000
	Income	1	0.002	0.966
	SRI	1	6.109	0.014
	EFT	1	8.203	0.005
	SRI x EFT	1	0.940	0.333
	Error	193		
Cigarette craving (QSU)	Corrected model	4	3.355	0.011
	Intercept	1	180.097	0.000
	Income	1	0.911	0.341
	SRI	1	5.932	0.016
	EFT	1	5.105	0.025
	SRI x EFT	1	0.228	0.633
	Error	194	3.355	0.011
Q_0 (Cigarette purchase task)	Corrected model	4	2.030	0.092
	Intercept	1	185.794	0.000
	Income	1	0.033	0.856
	SRI	1	4.808	0.030
	EFT	1	2.707	0.102
	SRI x EFT	1	0.010	0.919
	Error	193	2.030	0.092
Omax (Cigarette purchase task)	Corrected model	4	1.624	0.170
	Intercept	1	100.116	0.000
	Income	1	1.733	0.190
	SRI	1	1.731	0.190
	EFT	1	2.082	0.151
	SRI x EFT	1	0.229	0.633
	Error	193	1.624	0.170
Pmax (Cigarette purchase task)	Corrected model	4	0.226	0.923
	Intercept	1	22.689	0.000
	Income	1	0.328	0.567
	SRI	1	0.095	0.758
	EFT	1	0.033	0.856
	SRI x EFT	1	0.378	0.540

	Error	193	0.226	0.923
Affect (PANAS)	Corrected model	4	26.113	0.000
	Intercept	1	31.631	0.000
	Income	1	5.289	0.023
	SRI	1	79.189	0.000
	EFT	1	3.584	0.060
	SRI x EFT	1	1.914	0.168
	Error	194	26.113	0.000

AUC Area under the curve; *QSU* Questionnaire on Smoking Urges—Brief; *CPT* Cigarette purchase task; *PANAS* Positive and Negative Affect Scale
